# Novel cold-adapted raw-starch digesting α-amylases from *Eisenia fetida*: Gene cloning, expression, and characterization

**DOI:** 10.1016/j.btre.2021.e00662

**Published:** 2021-08-17

**Authors:** Kana Tsukamoto, Shingo Ariki, Masami Nakazawa, Tatsuji Sakamoto, Mitsuhiro Ueda

**Affiliations:** Graduate School of Life and Environmental Sciences, Osaka Prefecture University, Osaka, 599-8531, Japan

**Keywords:** *Eisenia fetida*, Raw-starch-digesting enzyme, Cold-adapted enzyme, Αlpha-amylase, Glycoside hydrolase family 13

## Abstract

We identified the raw-starch-digesting α-amylase genes a earthworm *Eisenia fetid α amylase I* and *II* (*Ef-Amy I* and *Ef-Amy II*). Each gene consists of 1,530 base pairs (bp) that encode proteins of 510 amino acids, as indicated by the corresponding mRNA sequences. Ef-Amy I and II showed an 89% amino acid identity. The amino acid sequences of Ef-Amy I and II were similar to those of the *α*-amylases from porcine pancreas, human pancreas, *Tenebrio molitor, Oryctolagus cuniculus*, and *Xenopus (Silurana) tropicalis*. Each gene encoding mature *Ef-Amy I* and *II* was expressed in the GS115 strain of *Pichia pastoris*. The molecular masses of the recombinant Ef-Amy I and II were 57 kDa each, and catalytically important residues of α-amylases of the GH family 13 were conserved in both proteins. These amylases exhibited raw-starch-digesting activity at 4 °C. The substrate specificities of rEf-Amy I and II were dissimilar. rEf-Amy I and II were shown to be active even in 40% ethanol, 4 M NaCl, and 4 M KCl.

## Highlights

• There have been few reports about gene cloning and expression of α-amylases from *E. fetida*.
• Ef-Amy I and II were shown to 89% identity of amino acid sequences.
• The catalytically important residues of α-amylase of GH family 13 were conserved in Ef-amy I and II.
• The substrate specificities of rEf-Amy I and II were dissimilar.
• It found that rEf-Amy I and II could be possible use for simultaneous saccharification and fermentation process.

## Graphical abstract

**Figure:**
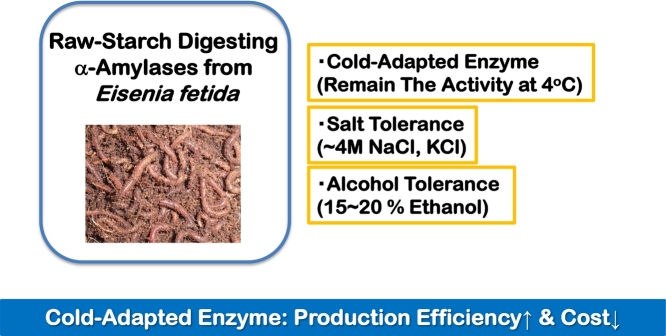

## 1 Introduction

Earthworms can be found all over the world, especially in temperate and tropical regions, where there is plenty of moisture in the ground. Earthworms are divided into 23 families with over 700 genera and more than 7000 species. They range from 25 to 1800 mm in length and are found seasonally at all depths in soil [1], where they represent the largest component of animal biomass and are recognized as ecosystem engineers with an excellent potential for being a partner to humans in regulating critical ecosystem functions. The importance of earthworms in supporting the soil structure, organic matter processing and nutrient cycling has long been acknowledged. In recent years, the use of earthworms in waste degradation has spurred interest in the processing of large quantities of waste materials. Earthworms degrade various organic matter. We found activities of glycoside hydrolases (GHs), such as cellulase, amylase, mannanase, and chitinase, in the crude enzyme solution of *Eisenia fetida* [2], [3], [4], [5]. To hydrolyze carbohydrates such as cellulose, starch, mannan, and chitin, the earthworm secretes digestive juices to the intestinal tract. In previous studies, we purified and characterized raw-starch-digesting amylases, β−1,4-glucanase, and a β−1,3-glucanase from *E. fetida* earthworms [3, 5, 6]. In addition, genes encoding endo-β−1,4-glucanase (Ef-EG2), mannanase (Ef-Man), and chitinase (Ef-Chi) were cloned and heterologously expressed in *Pichia pastoris*. The endo-β−1,4-glucanase, mannanase, and α-amylases from *E. fetida* are cold-adapted enzymes. The crystal structures of Ef-EG2, Ef-Man, and Ef-Amy I were solved [4, 7, 8]. The negatively charged amino acids Asp-and Glu-of Ef-EG2 occupy over two-thirds of the accessible surface area [7]. Acidic amino acids on the surface of the protein were correlated with low-temperature adaptation [9]. In a previous paper, we clarified the crystal structural of α-amylase I from *E. fetida* (Ef-Amy I) [8]. *α*-Amylases are GHs that degrade *α*−1,4 glycoside linkages in starch, glycogen, and related *α*-glucans. Ef-Amy I has been classified as belonging to the glycoside hydrolase family 13 with a typical (*β/α*)_8_-barrel containing two aspartic acids (D213, D316) and one glutamic acid (E249) that play essential roles in catalysis [8]. Ef-Amy I has structural similarities to mammalian α-amylases, including the porcine pancreatic and human pancreatic α-amylases. The raw starch-digesting α-amylases from *E. fetida* are endoenzymes that catalyze the breakdown of raw starch to glucose and maltooligosaccharides [2]. However, few studies have reported the structure and function of cold-adapted α-amylases from Annelida earthworms.

In this study, to understand the function of novel raw-starch-digesting α-amylases from *E. fetida*, we cloned and expressed Ef-Amy I and Ef-Amy II.

## 2 Materials and methods

### 2.1 Chemicals

Starch from potato, wheat, maize, amylopectin from maize, short-chain amylose (Amylose A), and long-chain amylose (Amylose B) were obtained from Nakalai Tesque, INC. (Kyoto, Japan). Starch from sweet potato was purchased from Wako Pure Chemicals Industries (Osaka, Japan). The rice starch, maltose (Mal), maltotriose (Mal3), maltotetraose (Mal4), and maltopentaose (Mal5) were obtained from Sigma-Aldrich, Japan (Tokyo, Japan). Beta-limit dextrin and maltohexaose (Mal6) were obtained from Megazyme Ltd. (Bray, Ireland). All other chemicals that were used for the reagents were of molecular biology grade.

### 2.2 Isolation of total RNA and cDNA synthesis

*E. fetida* earthworms were obtained from Nagane Industry (Sapporo, Japan), and only those that were nearly the same age were used in the experiments. The earthworms were washed, kept on wet filter papers, and starved for 24 h at 20 °C. Next, they were freeze-dried and grinded to a fine powder using a mortar and pestle. Total RNA was extracted from the freeze-dried worm powder using Isogen II (Nippon Gene, Japan) according to the manufacturer's instruction. First-strand cDNA was synthesized using an oligo(dt)17 adapter primer (GGCCACGCGTCGACTAGTACTTTTTTTTTTTTTTTTT) and Superscript III reverse transcriptase (Invitrogen, USA), according to the manufacturer's instructions.

### 2.3 cDNA cloning of α-amylase genes and construction of expression plasmids

We constructed an expressed sequence tag (EST) library containing approximately 70,000 contigs from *E. fetida* mRNA (data not shown). We found the α-amylase I (*Ef-Amy I*, contig no. c7174) and II (*Ef-Amy II*, contig no. c12319) genes in the EST library. We conducted cDNA cloning of the α-amylase genes on the basis of each mRNA sequence of contig no. c7174 and c12319. We had previously reported on the cloning and expression of *Ef-Amy I* in *P. pastoris* [8]. In this paper, we describe the cloning and expression *Ef-Amy II*.

Forward (5′- ATGTTTGGAATTCTTGTGACGCT-3′) and reverse (5′-GACGTGTATGGCAATCACGGGATCCT-3′) primers for each polymerase chain reaction (PCR) were synthesized to the regions corresponding to amino acid residues 1–8 and 498–506 of precursor *Ef-Amy II*. Each PCR was performed in a reaction mixture (20 μL) containing *E. fetida* cDNA, 0.5 μM of each primer, and 10 μL Takara PrimeSTAR HS polymerase (Takara Bio, Kyoto, Japan). The cycling parameters were the following: 35 cycles at 98 °C for 10 s, 55 °C for 5 s, and 72 °C for 120 s. The resulting 1.5-kb DNA fragment of each was cloned into the pGEM-T-easy vector (Promega, Fitchburg, WI, USA) according to the manufacturer's instructions. The nucleotide sequences of the amplified fragments were confirmed by sequencing.

To construct the expression vectors, forward (5′-GGCTGAAGCTGAATTCCAATACTTTGGAAGCTACT-3′, *Eco*RI site underlined) and reverse (5′-GAGTTTTTGTTCTAGAAAGACGTGTATGGCAATCACGG-3′, *Xba*I site underlined) primers were synthesized to regions corresponding to amino acid residues 18–24 and 500–506 of *Ef-Amy II*. The PCR reaction mixture (50 μL Prime STAR buffer) contained *E. fetida* cDNA, 0.25 μM of each primer, 200 μM of each dNTP, and 1.25 U Takara Prime STAR DNA polymerase (Takara Bio). The cycling parameters were as follows: 35 cycles at 98 °C for 10 s, 55 °C for 5 s, and 72 °C for 120 s. The resulting 1.5-kb DNA fragment of each was cloned into *Eco*RI and *Xba*I of the expression vector pPICZαA, according to the manufacturer's instructions for the In-Fusion HD Cloning Kit. The nucleotide sequence of the amplified fragments was confirmed by sequencing.

### 2.4 Expression and purification of the recombinant enzymes Ef-amy I and II (rEf-Amy I and Ef-amy II)

The expression plasmids pPICZαA-Ef-Amy I and pPICZαA-Ef-Amy II were linearized by *Sac*I and transformed into *P. pastoris* GS115 cells by electroporation. We previously expressed Ef-Amy I in *P. pastoris* [8]. Ef-Amy II was expressed using almost the same methods used to express Ef-Amy I. The nucleotide sequences of Ef-Amy I and II were optimized to overexpress in *P. pastoris* (data not shown). Cells harboring each plasmid were spread on 1% yeast extract, 2% peptone, 2% dextrose, 1 M sorbitol, 1.5% agar (YPDS) medium containing 100 μg/mL Zeocin™ and then incubated at 28 °C for 2∼4 days. Colonies were collected and each one was spread onto a YPDS plate containing 100, 500, 1000, or 2000 μg/mL of Zeocin™. Colonies that grew in the presence of a high concentration of Zeocin™ were then selected. The selected colonies were cultured in a 1-L Erlenmeyer flask containing 200 mL BMGY medium (1% yeast extract, 2% peptone, 100 mM potassium phosphate pH 6.0, 1.34% YNB, 4 × 10^−5^% biotin, 1% glycerol) at 28 °C. The culture media was centrifuged at 8200 × *g* for 10 min and the resulting cell pellets were resuspended in BMMY medium (1% yeast extract, 2% peptone, 100 mM potassium phosphate pH 6.0, 1.34% YNB, 4 × 10^−5^% biotin, 0.5% methanol). The cell suspension was then added to 800 mL of BMMY medium and grown at 17 °C for 7 days, during which 0.5% methanol was added daily.

After cultivation of rEf-Amy I and II, each culture medium was centrifuged at 10,000 × *g* for 30 min at 4 °C before the supernatant was recovered. rEf-Amy I and II were purified using the same procedure. The culture filtrate was concentrated using a cellulose tube with polyethylene glycol 6000 and was dialyzed against 20 mM Tris–HCl buffer (pH 8.0) overnight at 4 °C with stirring. After dialysis of the concentrated culture filtrate, the enzyme solution was loaded onto a DEAE-Toyopearl 650 M column (2.5 cm [inner diameter)] × 10 cm; Toso, Tokyo, Japan), which was equilibrated with 20 mM Tris–HCl buffer (pH 8.0). The enzyme bound to the gel was eluted with 20 mM Tris–HCl buffer (pH 8.0) buffer containing 1.0 M NaCl at a flow rate of 1.0 mL min^−1^. The protein contents in the elution fractions were measured by monitoring the absorbance at 280 nm. The active fractions were dialyzed with a cellulose tube in 20 mM Tris–HCl buffer (pH 8.0). Ef-Amy I and II were loaded onto a HisTrap FF column (column volume: 1 mL; GE Healthcare, Little Chalfont, Buckinghamshire, UK) equilibrated with 20 mM Tris–HCl buffer (pH 8.0) containing 0.5 M NaCl. The enzyme was eluted with a linear gradient of imidazole (0–500 mM) in 20 mM Tris–HCl buffer (pH 8.0) containing 0.5 M NaCl. The active fractions after HisTrap FF column chromatography were dialyzed with a cellulose tube in 20 mM Tris–HCl buffer (pH 8.0) and were used as the purified enzyme solution.

### 2.5 Enzyme assay and protein determination

The amylase activity was measured by determining the amount of reducing sugar released from soluble starch (Kanto Chemicals, Tokyo, Japan). The amount of enzymatic activity required to form the amount of reducing sugar that corresponds to 1 μmol of glucose per min of reaction time was regarded as one unit of enzymatic activity. The standard assay method involves a reaction mixture consisting of 0.4% soluble starch in 50 mM sodium acetate buffer (rEf-AmyI: pH 5.5; rEf-AmyII: pH 5.0) and an enzyme to give a final volume of 0.3 mL. After incubation for 20 min at 37 °C, the amount of reducing sugar contained in the sample was determined according to the method of Somogyi-Nelson [10]. The protein concentrations of rEf-Amy I and II were calculated with absorbance at 280 nm and the protein extinction coefficient according to the method of Gill and von Hippel [11].

### 2.6 Effects of temperature and pH on enzymatic activities

The enzymatic activities were measured by the standard assay method with 0.4% soluble starch used as the substrate at various temperatures and pH levels. The effect of temperature on enzymatic activity was examined at 10–80 °C. The buffers used were 0.1 M sodium acetate (pH 4.0 to 6.0), 0.1 M KH_2_PO_4_-K_2_HPO_4_ (pH 6.0 to 8.0), 0.1 M Tris–HCl (pH 7.0 to 9.0), and 0.1 M NaHCO_3_—Na_2_CO_3_ (pH 9.0 to 10.0).

### 2.7 Effects of temperature and pH on enzyme stability

To measure the thermal stability, each purified enzyme (13.8 units/mL) was incubated in 0.1 M KH_2_PO_4_-K_2_HPO_4_ buffer (pH 8.0) for 30 min at various temperatures in the range of 10–80 °C. After incubation, the remaining activities were measured under standard assay conditions. The pH effect on enzyme stability was determined by incubating the enzymes (13.8 units/mL) for 24 h at 4 °C in 0.1 M of each of the following buffers: sodium acetate (pH 4.0 to 6.0), KH_2_PO_4_-K_2_HPO_4_ (pH 6.0 to 8.0), Tris–HCl (pH 7.0 to 9.0), and NaHCO_3_—Na_2_CO_3_ (pH 9.0 to 11.0).

### 2.8 Molecular mass

The molecular mass was estimated by sodium dodecyl sulfate polyacrylamide gel electrophoresis (SDS-PAGE) following the method of Laemmli [12] with Precision Plus Protein standard (Bio-Rad Co., CA, USA). Protein bands were detected by staining with Coomassie Brilliant Blue R-250.

### 2.9 Substrate specificities

The activities of rEf-Amy I and II were tested using soluble starch, amylopectin, beta-limit dextrin, short-chain amylose, and long-chain amylose. In each case, the breakdown of starch was assayed by the production of reducing sugars and measured as described above.

### 2.10 High performance liquid chromatography (HPLC) analysis of hydrolysis products from maltooligosaccharides

To investigate cleavage patterns from the hydrolysis products of the purified enzymes, 5 mM maltooligosaccharide substrates (Mal3, Mal4, and Mal5) were dissolved in 50 mM sodium acetate buffer (rEf-AmyI: pH 5.5; rEf-AmyII: pH 5.0), and aliquots of the enzyme solution were added to 200 μL of each substrate solution. Enzyme reactions were performed at 37 °C for various times, and portions of the reaction mixture were then withdrawn and mixed with the same volume of chilled acetonitrile (−20 °C) to terminate the reaction. The resulting solutions were then applied to a Sugar-D column (4.6 × 250 mm; Nakalai Tesque, INC.) and were eluted with 70% acetonitrile at a flow rate of 1.0 mL/min. Substrates and products were monitored with an RI detector (Jasco Co., Tokyo, Japan).

### 2.11 Effect of metal ions on the enzymatic activities

The remaining activities were determined with the standard assay using soluble starch following the pre-incubation of each enzyme (13.8 units/mL) in 0.1 M Tris–HCl (pH 8.0) containing various metal ions at 4 °C for 24 h. The concentration of metal ions and EDTA were used in 1 mM. All the metal ions were added as chloride salts.

### 2.12 Effects of ethanol concentration on the enzymatic activities

The enzymatic activities were measured by the standard assay method with 0.4% soluble starch used as the substrate at various concentrations of ethanol. The concentrations of ethanol were 0%–40%.

### 2.13 Effects of chloride compounds on enzyme stabilities

The remaining activities were determined with the standard assay using soluble starch following the pre-incubation of each enzyme (13.8 units/mL) in 0.1 M Tris–HCl (pH 8.0) containing KCl or NaCl at 4 °C for 24 h. The concentrations of NaCl and KCl were 0–4 M.

### 2.14 Nucleotide sequence accession number

The *E. fetida Ef-Amy II* mRNA data reported in the present paper have been submitted to the DDBJ, EMBL, and NCBI databases under the accession number LC594654.

## 3 Results and discussion

### 3.1 Cloning and sequencing of the Ef-Amy I and II genes

**Fig. 1:**
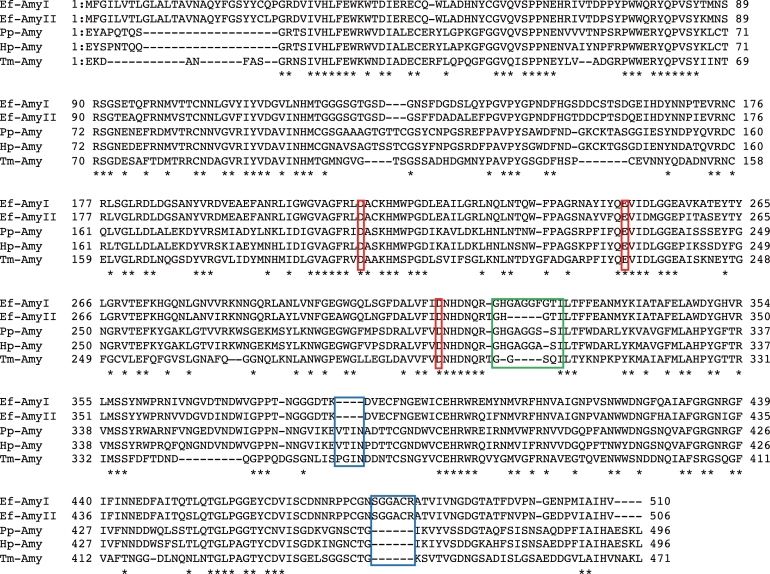
Sequence alignment of α-amylases I and II from *E. fetida, α*-amylases from porcine pancreas (PP-Amy, 55% and 55%, 1PIG_A), human pancreas (Hp-Amy, 54% and 54%, 4W93_A), and *Tenebrio molitor* (Tm-Amy, 47% and 48%,1JAE_A). The percentages indicated in parentheses represent the sequence identities with the amino acid sequences of Ef-Amy I and II from *E. fetida*. The asterisks indicatethe conserved amino acids among the α-amylases. Red boxes indicate catalytic amino acids.The Gly-rich loops is indicated by a green box. The deleted and inserted loops in Ef-Amy I and II are indicated by blue boxes.

The α-amylases were already purified from the crude extract of *E. fetida* [3]. We also reported that Ef-Amy I was expressed in *P. pastoris* [8]*.* The present study shows that Ef-Amy II is functionally expressed in the earthworm species *E. fetida.* The *Ef-Amy II* gene was determined to be 1530 bp and to encode a protein of 510 amino acids; its mRNA sequence was deposited in the GenBank database (LC594654). The amino acid sequences of Ef-Amy I (BAV13234.1) and II (LC594654) were similar to those of the *α*-amylases from porcine pancreas (Pp-Amy, 55% and 55%, 1PIG_A), human pancreas (Hp-Amy, 54% and 54%, 4W93_A), *Tenebrio molitor* (Tm-Amy, 47% and 48%, 1JAE_A), *Oryctolagus cuniculus* (55% and 55%, XP_002715870), and *Xenopus (Silurana) tropicalis* (Xt-Amy, 56% and 56%, XP_002938902). Given that animal *α*-amylases belong to the GH family 13, Ef-Amy I and II may also belong to this enzyme family. Pp-Amy, Hp-Amy, and Xt-Amy are included in the subfamily GH13_24 [13]. In addition, all catalytically important residues of *α*-amylases from the GH family 13 were conserved in Ef-Amy I and II (D213, D316, and E249), as shown in Pp-Amy, Hp-Amy, and Tm-Amy (Fig. 1). It was suggested that D213 is a nucleophile residue and E249 is an acid/base catalyst [14]. The N-terminal domain (E18–G416) consists of an *α/β*-fold TIM barrel, and the C-terminal domain (N417–V510) forms a *β*-barrel [8]. The binding sites for Ca^2+^ ions in Ef-Amy I are located at the same positions in the protein as in Pp-Amy and Hp-Amy [8]. The amino acid residues N118, R174, D183, and H217 in relation to Ca^2+^ binding of animal α-amylases were conserved in Ef-Amy I and II.

There is no starch binding domain in rEf-Amy I, based on a structural study [8]. rEf-Amy II also does not contain the starch-binding domain. It was reported that Y276 of Hp-Amy plays an important role in starch binding to the protein surface [15]. Y292 is conserved in rEf-Amy II, but Y292 in rEf-Amy I changed to N292 (Fig. 1). The difference in the amino acid sequence may affect the starch-binding ability. To clarify the starch-binding ability of rEf-Amy II, we intend to solve the structure of Ef-Amy II.

Hirano et al. reported that a glucose unit of Mal4 has a stacking interaction with Trp401 in the Ef-Amy I structure [8]. The two loops surrounding the Mal4-binding site of Ef-Amy I showed structural differences between the loops in Pp-Amy and Hp-Amy. The structures of starch recognized by Ef-Amy I may be different from those recognized by Pp-Amy and by Hp-Amy. This might also be the case for Ef-Amy II. To understand the function of the surface-binding site of rEf-Amy I and II, we intend to conduct several mutational studies.

### 3.2 Phylogenetic analysis of Ef-Amy Iand II

**Fig. 2:**
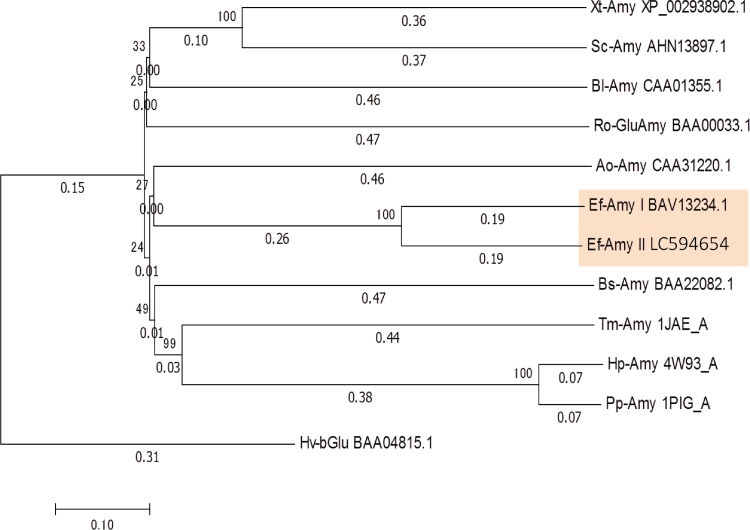
Phylogenetic tree of α-amylases from *E. fetida* and other species from the GH family 13. The enzyme names and accession numbers are Ef-Amy I (BAV13234.1), Ef-Amy II (LC594654), *α*-amylases from porcine pancreas (Pp-Amy, 1PIG_A), human pancreas (Hp-Amy, 4W93_A), *Tenebrio molitor* (Tm-Amy, 1JAE_A), *Bacillus* sp. (Bs-Amy, BAA22082.1), *B. licheniformis* (Bl-Amy, CAA01355.1), *Aspergillus oryzae* (Ao-Amy, CAA31220.1), *Siganus canaliculatus* (*Sc*-Amy, AHN13897.1), *Xenopus (Silurana) tropicalis* (Xt-Amy, XP_002938902), glucoamylase from *Rhizopus oryzae* (Ro-Amy, BAA00033.1), and b-amylase from *Hordeum vulgare* subsp. *vulgare* (Hv-Amy, BAA04815.1). We used Clustal Omega (http://www.ebi.ac.uk/Tools/msa/clustalo/) to construct the phylogenetic tree.

Phylogenetic analyses were performed based on the amino acid sequence homologies of Ef-Amy I, Ef-Amy II, and α-amylases from other species. We determined that Ef-Amy I and II were most close to *α*-amylases from *Aspergillus oryzae* (Fig. 2).

### 3.3 Expressions of Ef-Amy I and II genes and purification of recombinant proteins

**Fig. 3:**
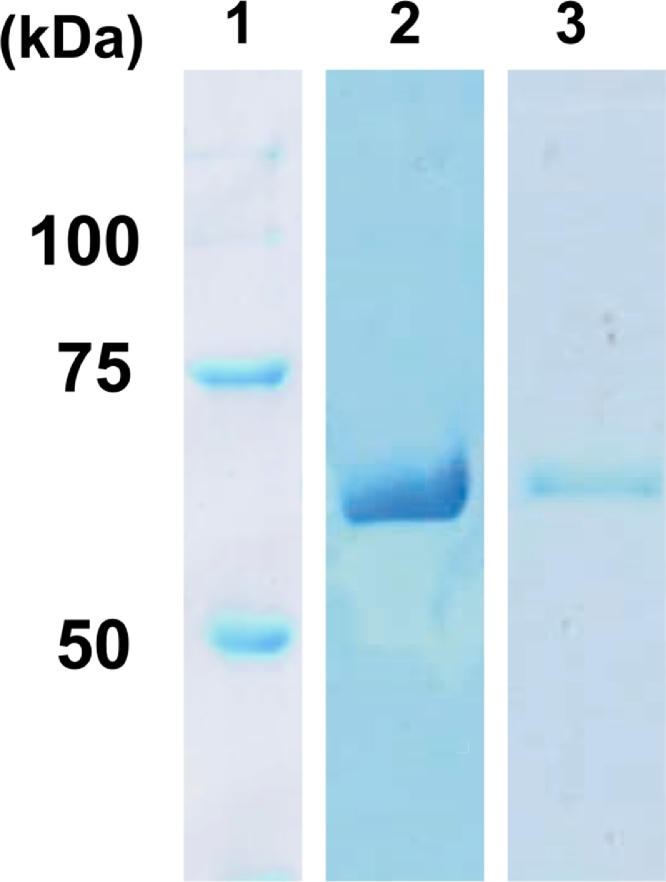
SDS–PAGE of the recombinant purified Ef-Amy I and II. The Precision Plus Protein All Blue Standard was used as a marker (Bio-Rad, USA). 1: marker, 2: recombinant purified Ef-Amy I, 3: recombinant purified Ef-Amy II.

The mature active form of Ef*-*Amy II was expressed in *P. pastoris* GS115 similarly to Ef-Amy I [8]. The present rEf-Amy I and II were purified from *P. pastoris* GS115 harboring pPICZαA-Ef-Amy I and II, respectively. The molecular mass of each protein was estimated to be 57 kDa using SDS-PAGE (Fig. 3).

### 3.4 Effects of temperature and pH on the activity and stability of rEf-Amy I and II

**Fig. 4:**
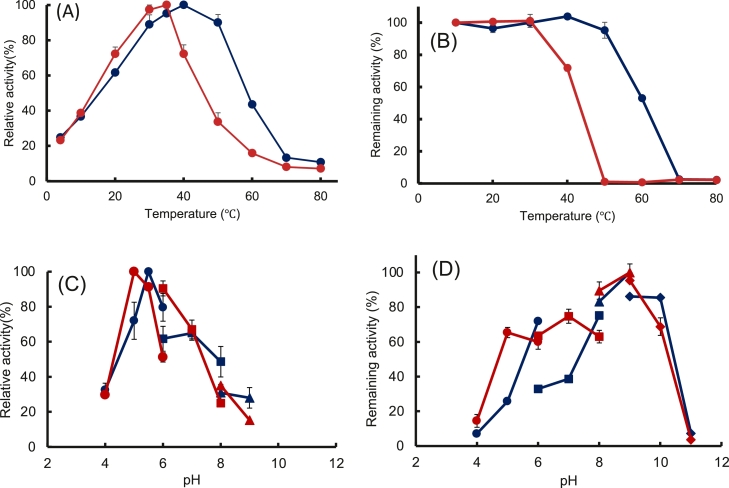
Functional properties of purified rEf-Amy I and II. All reactions were conducted with each purified enzyme using soluble starch as the substrate. The dark blue line represents rEf-Amy I, and the red line represents rEf-Amy II. (A) Effects of temperature on enzymatic activities measured from 0 to 80 °C. (B) Effects of temperature on enzyme stability. Assays were performed at 37 °C after 30-min incubation in 20 mM sodium acetate buffer (pH 5.0) at 10–80 °C. (C) Effects of pH levels on enzymatic activities at 37 °C in the following 0.1 M buffers: sodium acetate (pH 4.0‒6.0), KH_2_PO_4_-K_2_HPO_4_ (pH 6.0‒8.0), and Tris–HCl (pH 8.0‒9.0). (D) Effects of pH levels on enzyme stability. Assays were conducted at 37 °C (rEf-Amy I: pH 5.5; rEf-Amy II: 5.0) after 24-h incubation in the following 0.1 M buffers: sodium acetate (pH 4.0‒6.0), KH_2_PO_4_-K_2_HPO_4_ (pH 6.0‒8.0), Tris–HCl (pH 8.0‒9.0), and carbonate-bicarbonate (pH 9.0‒11.0).

The properties of rEf-Amy I and II at various temperatures and pH values were determined in enzyme assays using soluble starch as the substrate. The optimal temperatures for rEf-Amy I and II activities were 40 °C and 35 °C, respectively (Fig. 4A), and these enzymes were stable up to 50 °C and 30 °C, respectively (Fig. 4B). It was suggested that rEf-Amy I is a moderately thermostable α-amylase, as those of *Bacillus subtilis* 65, *Bacillus* YX-1, *Streptomyces* sp. E2248, and *Cryptococcus* sp. S-2 [20], [21], [22], [23]. rEf-Amy I and II were shown to be active at temperatures as low as 20 °C, and some activities at 4 °C. We also observed that rEf-Amy I and II had raw-starch-digesting activity at the levels of 0.85 and 0.79 units/mg protein at 4 °C, respectively. rEf-Amy I and Amy II are also cold-adapted α-amylases as the endogenous Amy I and II from *E. fetida* [2]*.*

The optimum pH levels of rEf-Amy I and II were 5.5 and 5.0, respectively, and their activities were stable between pH 8.0 and 10.0 and pH 8.0 and 9.0, respectively (Figs. 4C and 4D). Animal α-amylases from *Homo sapiens* (pH 6.9), rat (pH 6.9), *Haliotis discus* (pH 6.5), and *Mytilus galloprovincialis* (pH 6.5) have optimum pH values of 6.5 ∼ 6.9 [16], [17], [18], [19]. We found that the optimum pHs of rEf-Amy I and II were different for those of the abovementioned animal enzymes.

### 3.5 Hydrolysis products of rEf-Amy Iand II

**Fig. 5:**
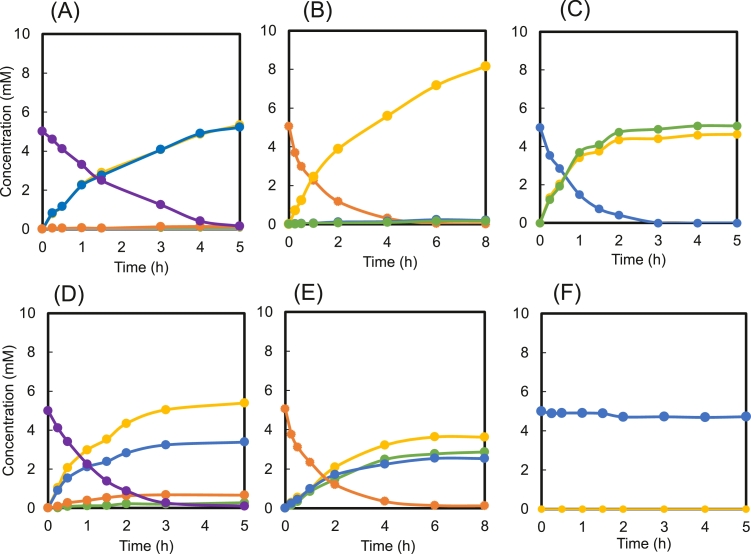
HPLC analysis of hydrolysis products from maltooligosaccharides (Mal3, Mal4, and Mal5) by rEf-Amy I (A, B, C) and rEf-Amy II (D, E, F). The enzymatic degradation products of (A) and (D): Mal_5_; (B) and (E): Mal4; (C) and (E): Mal3 were detected by HPLC. Lines: glucose (yellowish green–olive line), Mal (yellow line), Mal3 (blue line), Mal4 (yellowish brown line), and Mal5 (purple line). Data are presented as means of triplicate measurements.

To identify modes of action of purified rEf-Amy I and II, enzymatic activity assays were conducted with maltooligosaccharides of various lengths, including Mal, Mal3, Mal4, and Mal5, and buffer aliquots were collected over time and analyzed using high-performance liquid chromatography (Fig. 5). In the case of Ef-Amy I, the major hydrolysis products from Mal5 were Mal and Mal3 (Fig. 5A), from Mal4 was Mal (Fig. 5B), and from Mal3 were Mal and glucose (Fig. 5C). On the other hand, the major hydrolysis products from Mal5 using Ef-Amy II were Mal and Mal3 (Fig. 5D), being Mal4 a minor hydrolysis product, and from Mal4 were Mal, Mal3_,_ and glucose (Fig. 5E). In contrast, Mal3 was not degraded (Fig. 5F). The hydrolysis products of rEf-Amy I and II were dissimilar (Fig. 5). A structural study using the loop deletion mutant of the human salivary α-amylase suggested that the Gly-rich loop is involved in the release of products from the active site [24]. Hirano et al. reported that the Gly-rich loop in Ef-Amy I may play an important role in recognizing substrates and inhibitors [8]. The Gly-rich loop in Ef-Amy I (G320-HGAGGFG327) is one amino acid longer than that in Hp-Amy (G319-HGAGGA325) and Pp-Amy (G319-HGAGGS325). However, there is no Gly-rich loop in Ef-Amy II. These findings suggest that the Gly-rich loop is involved in the hydrolysis of oligosaccharides by modulating the conformation of the glucose unit at the −2 subsite [8]. It has been proposed that the difference between the hydrolysis products for rEf-Amy I and II were caused by the difference in the Gly-rich loop (Fig. 5). It is reported that amylases belonging to GH 13 react the transglycosylastion [25]. It is indicated that Ef-Amy I and II may also react with transglycosylation. We intend to investigate the transglycosylation reaction of these two enzymes.

### 3.6 Substrate specificity

**Fig. 6:**
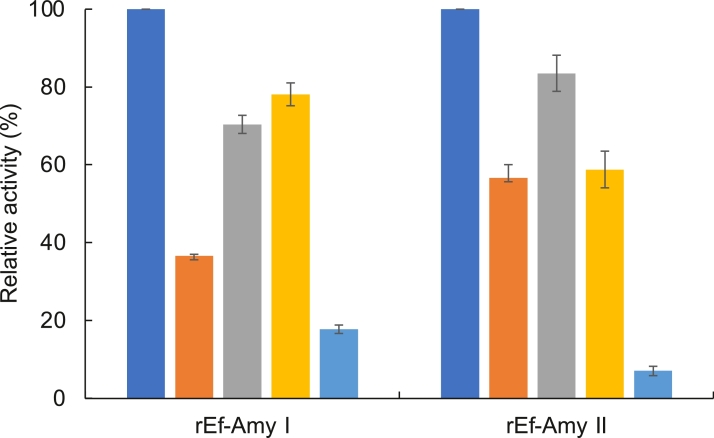
Substrate specificities of rEf-Amy I and II. Enzymatic activities were measured using the standard assay method with 0.4% of various substrates. Bars: soluble starch (blue bar), amylopectin (yellowish-brown bar), beta-limit dextrin (gray bar), amylose A (yellow bar), amylose B (light blue bar). Each data point represents the average of triplicate measurements. Error bars show standard deviation of measurements.

The enzyme rEf-Amy I was highly active against soluble starch, *β*-limit dextrin, and short-chain amylose, but had lower activity against amylopectin and long-chain amylose (Fig. 6). On the other hand, rEf-Amy II had higher activity against soluble starch, amylopectin, and *β*-limit dextrin, but had lower activity against long-chain amylose. It was considered that rEf-Amy I hydrolyzes substrates with *α*−1,4 carbohydrate bonds only, whereas rEf-Amy II hydrolyzes substrates with *α*−1,6 and *α*−1,4 carbohydrate bonds. The specific activity of rEf-Amy I and II were 174 and 65 units/mg of protein in the presence of a soluble-starch substrate, and 6.5 and 4.2 units/mg of protein in the presence of insoluble starch (raw starch). The specific activities of rEf-Amy I and II were different from those of the endogenous Amy I and II from *E. fetida* [2]. The reason for this difference is currently unclear. It was suggested that Ef-Amy I having a Gly-rich loop facilitated a reaction to the substrates in the active site compared to Ef-Amy II. Following digestion of Mal5 and Mal4 by rEf-Amy I and II, the specific activities of Mal5 and Mal4 were 34 and 7.1 mM/min/mg protein for rEf-Amy I and 8.4 and 3.2 mM/min/mg protein for rEf-Amy II during the early stage of hydrolysis. It was suggested that rEf-Amy I and II require at least five or more subsites for efficient hydrolysis. In agreement, crystallographic data of rEf-Amy I [8] and pancreatic *α*-amylase [26] indicate that these enzymes utilize more than five subsites for efficient hydrolysis.

### 3.7 Effects of metal ions and EDTA on the enzymatic activities

The effects of various metal ions and EDTA on the enzymatic activities are shown in Table 1. The enzymatic activity of rEf-Amy I was strongly inhibited by Ag^+^, Al^3+^ and Hg^2+^ ions, whereas that of rEf-Amy II was strongly inhibited by Al^3+^ and Hg^2+^ ions. The addition of Zn^2+^ ions resulted in moderate inhibition. In general, *α*-amylases are stabilized by Ca^2+^ ions in the architecture of the active site [27]. In this study, the addition of Ca^2+^ ions had no effect on the enzymatic activities. Hirano et al. reported that the binding sites for the Ca^2+^ ion in Ef-Amy I are the same as those in Pp-Amy and Hp-Amy [8]. The addition of EDTA did not influence the catalytic activity of Ef-Amy I, but that of Pp-Amy was completely inhibited in the presence of 125 μM of EDTA [28]. Hirano et al. suggested that the structure at the entrance of the Ca^2+^ ion-binding cavity affects the access of EDTA molecules to the Ca^2+^ ion-binding site. It is supposed that Ef-Amy II has also almost the same structure around the binding sites of Ca^2+^ ion.

**Table 1:** Effects of metal ions and EDTA on the enzyme activities.

	Remaining activity (%)	
Added substance	rEf-Amy I	rEf-Amy II
No addition	100	100
Ag⁺	10	59
Al³⁺	19	10
Ba²⁺	87	98
Ca²⁺	74	117
Cu²⁺	42	80
Hg²⁺	5	16
Mg²⁺	88	94
Mn²⁺	86	70
Zn²⁺	34	45
EDTA	127	85

The average values of triplicate measurements were used as values for each activity .

### 3.8 Effect of ethanol on the enzymatic activities

**Fig. 7:**
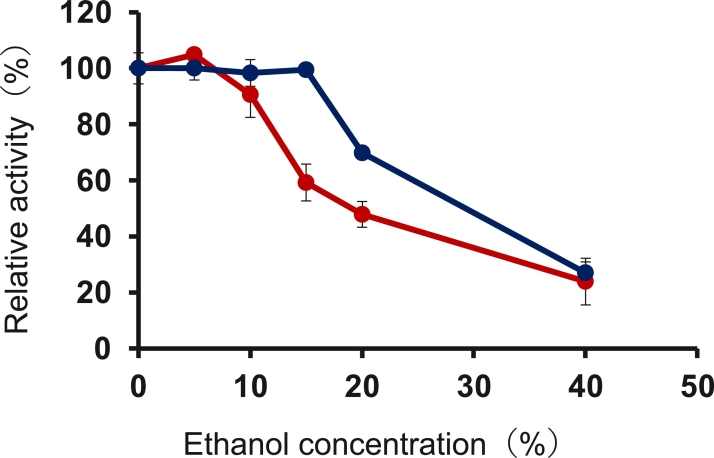
Effects of ethanol concentration on enzymatic activities. Enzymatic activities were measured using the standard assay method with 0.4% soluble starch as the substrate at various concentrations of ethanol (approximately 40% v/v). The dark blue line represents rEf-Amy I, and the red line represents rEf-Amy II. Each data point represents the average of triplicate measurements. Error bars show standard deviation of measurements.

rEf-Amy I showed high activity at the concentration of 15% ethanol, whereas rEf-Amy II showed a lower activity (60%) than rEf-Amy I (Fig. 7). rEf-Amy I and II maintain their enzymatic activities at the concentration of 40% ethanol. In simultaneous saccharification and fermentation (SSF) process, the ethanol concentration increased up to 15%∼20% [7, 29, 30]. It was found that rEf-Amy I and II could be used for this process.

### 3.9 Effect of chloride compounds on the enzymatic activities

**Fig. 8:**
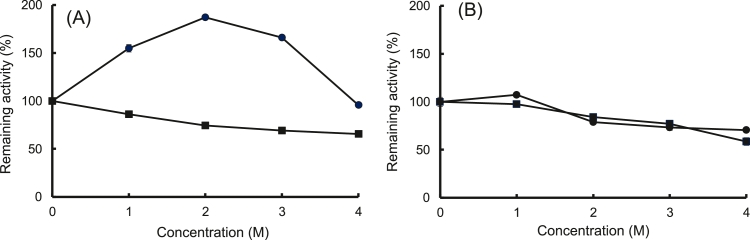
Effects of the KCl and NaCl concentrations on enzyme stabilities. Each enzyme was pre-incubated at 4 °C for 24 h in the final concentration of 0–4 M of NaCl or KCl. After pre-incubation, the remaining activities were measured using the standard assay method with 0.4% soluble starch used as the substrate. (A): rEf-Amy I, (B): rEf-Amy II. ●: NaCl, ■: KCl. Each data point represents the average of triplicate measurements. Error bars show standard deviation of measurements.

rEf-Amy I and II maintain their enzymatic activities at high concentrations (4 M) of NaCl and KCl (Fig. 8). It was found that rEf-Amy I and II are halophilic enzymes. It has been proposed that the Cl^−^ ion enhances catalytic activity [31, 32]. In particular, the activity of rEf-Amy I increased by approximately two-fold in the presence of 2 M NaCl (Fig. 8A). The binding stie for Cl^−^ in Ef-Amy I is located in the same position as in Pp-Amy and Hp-Amy [8]. However, it was reported that the activity of Hp-Amy was inhibited at a higher concentration of chloride (> 400 mM) [32]. We intend to elucidate the mechanism of salt tolerance of Ef-Amy I and II.

## 4 Conclusions

To our knowledge, there are few peports about gene cloning and expression of α-amylases from the Annelida earthworm *E. fetida*. The catalytic residues of the GH family 13 *α*-amylases were conserved in Ef-Amy I and II. Moreover, the major hydrolysis products of maltooligosaccharides by rEf-Amy I and II were dissimilar. The rEf-Amy I is a more thermostable enzyme than rEf-Amy II. The activities of rEf-Amy I and II were shown to occur at temperatures as low as 4 °C. rEf-Amy I and II showed high activity at the concentration of 15% ∼ 20% ethanol. Ef-Amy I and II may be used for the SSF process. We suggest that Ef-Amy II may have a partially different crystallographic structure than Ef-Amy I. In future studies, we intend to solve the structure of Ef-Amy II by using X-ray crystallography.

## Credit authorship contribution statement

Kana Tsukamoto: Writing - original draft, Data curation, Investigation. Singo Ariki: Data curation, Investigation, Masami Nakazawa: Supervision. Tatsuji Sakamoto: Supervision. Mistuhiro Ueda: Supervision, Conceptualization, Data curation, Writing - review & editing.

## Conflict of interest

The authors declare no conflict of interest.
